# Re-exposure to nicotine-associated context from adolescence enhances alcohol intake in adulthood

**DOI:** 10.1038/s41598-017-02177-2

**Published:** 2017-05-30

**Authors:** Dor Zipori, Yossi Sadot-Sogrin, Koral Goltseker, Oren Even-Chen, Nofar Rahamim, Ohad Shaham, Segev Barak

**Affiliations:** 1 School of Psychological Sciences, Tel Aviv University, Tel Aviv, Israel; 2 Sagol School of Neuroscience, Tel Aviv University, Tel Aviv, Israel

## Abstract

Alcohol and nicotine are the two most commonly-abused substances and are often used together. Nicotine enhances alcohol-drinking behaviors in humans and in animals, and was suggested to enhance the reinforcing properties of other reinforcers. Here, we show that nicotine-associated environment, rather than nicotine itself, enhances alcohol intake in rats. Adolescent rats received repeated intermittent injections of nicotine (0.4 mg/kg, i.p., 5 injections, every 3^rd^ day) or saline. The injection was paired with their home cage, or with the subsequent alcohol self-administration context. Rats were then trained to self-administer 20% alcohol. Nicotine given in the home cage did not alter subsequent alcohol intake. However, pairing nicotine with the operant chamber during adolescence led to a long-lasting increased alcohol self-administration in adulthood, compared to nicotine pre-treatment in other contexts. This effect persisted 3 months after nicotine cessation, in a relapse test after abstinence. Furthermore, re-exposure to the nicotine-associated context in adult rats led to a decrease in glial cell line-derived neurotrophic factor *(Gdnf)* mRNA expression in the ventral tegmental area, an effect that leads to increased alcohol consumption, as we ﻿have previously reported. Our findings suggest that retrieval of nicotine-associated contextual memories from adolescence may gate alcohol intake in adulthood, with a possible involvement of GDNF.

## Introduction

Alcohol and nicotine are the two most commonly-abused substances in the general population, and are frequently used together^1^. Epidemiological studies suggest that heavy use of one of these substances is often predictive of heavy use of the other substance. Specifically, alcohol use disorders are more common among smokers, who are 50% more likely to drink regularly than non-smokers^2^. Likewise, tobacco smoking prevalence among heavy drinkers is 2–3 times higher than in the general population^3^. In the US, 7% of adolescents, and 35% of young adults are tobacco users^4^, and the likelihood for alcohol abuse is increased among early-onset tobacco smokers^5^. These associations are thought to reflect the capacity of alcohol and nicotine to enhance the motivation to obtain the other substance, as supported by studies in humans^6, 7^ and rodents^8, 9^.

For example, repeated nicotine treatment increases alcohol self-administration in rodents^10–13^, whereas nicotinic antagonists^14, 15^ or partial agonists^16^ reduce alcohol self-administration. Moreover, nicotine was shown to enhance relapse to alcohol seeking in a self-administration reinstatement rat model^12, 17^.

Nicotine has also been shown to enhance the self-administration and the rewarding properties of psychostimulants, such as cocaine^18–20^, amphetamine^21^, or nicotine itself  ^22, 23^. However, when animals are first pre-exposed to nicotine, and are then subjected to drug self-administration, results are mixed^21, 23, 24^.

Interestingly, the environmental context of nicotine pre-exposure has been suggested to play a critical role in nicotine’s effects on subsequent psychostimulant self-administration^18, 21, 23^. Specifically, rats’ self-administration of stimulants in a context previously associated with nicotine resulted in enhanced intake of cocaine^18^, amphetamine^21^ and nicotine^23^. However, nicotine exposure in the home cage did not always affect drug consumption^22–24^. These findings raise the possibility that nicotine-paired contextual cues, rather than the dynamic pharmacological effects of nicotine itself, enhance subsequent drug self-administration.

It is plausible that nicotine, or re-exposure to nicotine-related context, affects alcohol intake by affecting the expression of genes that control alcohol consumption^25^. Thus, within the mesolimbic ‘reward’ system, which includes dopaminergic projections from the ventral tegmental area (VTA) to the nucleus accumbens (NAc), co-administration of nicotine and alcohol into the VTA was shown to cause downregulation in the expression of glial cell line-derived neurotrophic factor (*Gdnf*)^26^. Recently, we showed that downregulation of *Gdnf* in the VTA or NAc by short-hairpin RNA (shRNA) led to increased alcohol consumption and relapse^27, 28^. Thus, it is plausible that re-exposure to nicotine-associated cues facilitates alcohol seeking and intake by causing neuroadaptations that trigger the reward system, such as alterations in the expression of *Gdnf*.

As the first exposure to nicotine in humans typically occurs in adolescence^4^, here we determined the effects of re-exposure to the context associated with nicotine during adolescence on operant alcohol self-administration in adulthood. Then, we assessed whether re-exposure to the nicotine context alters the mesolimbic expression of *Gdnf*, which controls alcohol-drinking behaviors.

## Results

### Adolescent nicotine pre-treatment enhances operant alcohol self-administration in adulthood only when nicotine is paired with the operant chamber

#### Experiment 1

**Figure 1:**
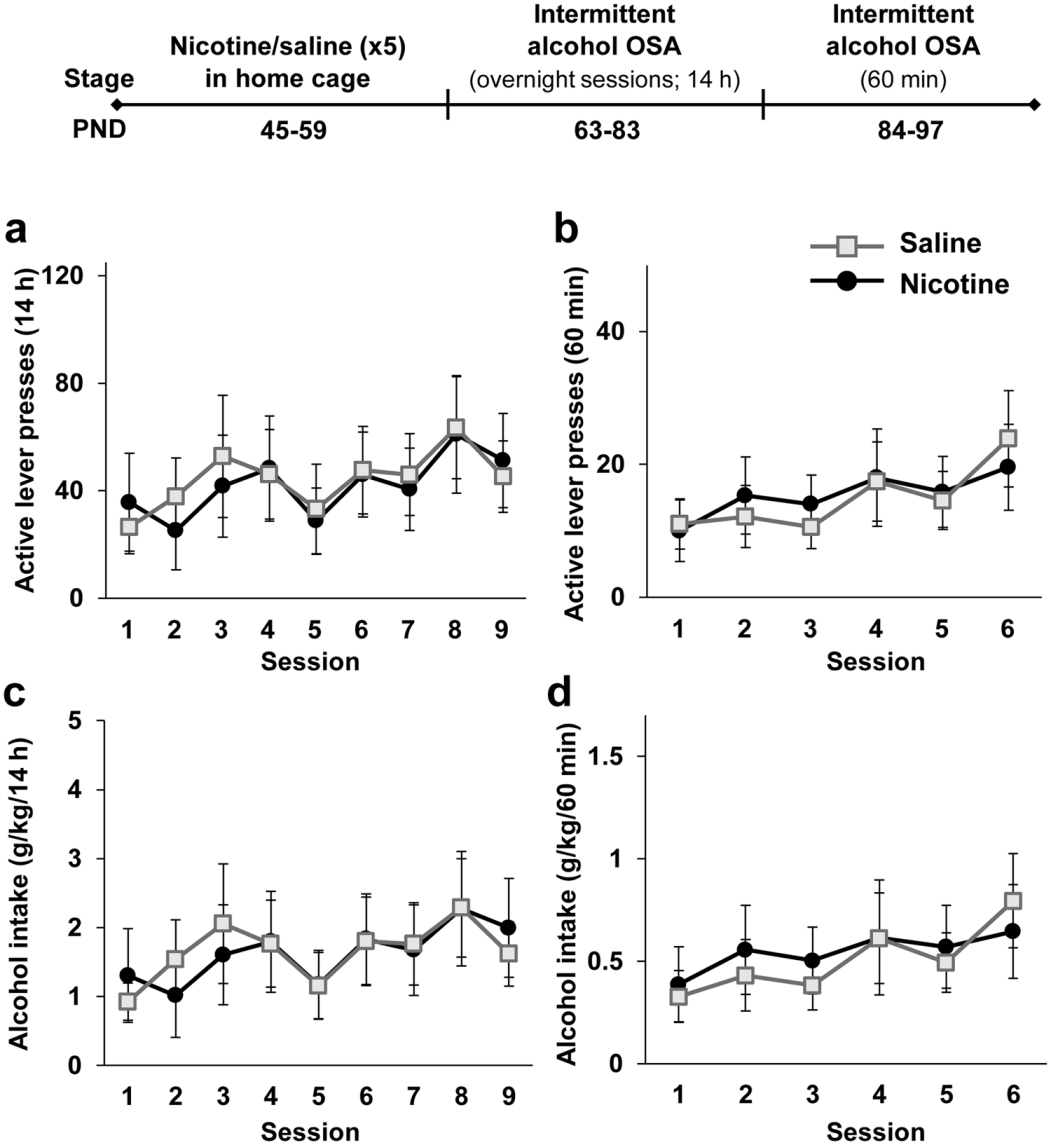
Adolescent nicotine pre-treatment in the home cage does not enhance operant alcohol self-administration in adulthood. Rats were treated with 5 nicotine or saline injections on PND 45–59, and remained in the home cages. On PND 63 rats began intermittent overnight operant alcohol self-administration (OSA) training three days a week (fixed ratio 1; FR1) for 3 weeks. On PND 84, rats began intermittent operant training (FR1) of 60 min, three days a week for 2 weeks. (**a–d**) Means ± SEM of the number of active lever presses (**a** and **b**) or alcohol intake normalized to body weight (**c** and **d**) during the overnight (**a** and **c**) or 60-min (**b** and **d**) sessions. Mixed-model ANOVA; Overnight sessions: A main effect of Session for active-lever presses [F(8,96) = 2.61, p < 0.05] and for alcohol intake [F(8,96) = 2.05, p < 0.05]; 60-min sessions: A main effect of Session for active-lever presses [F(5,60) = 5.15, p < 0.05] and for alcohol intake [F(5,60) = 4.67, p < 0.05]; No significant main effect of Pre-treatment and no significant Pre-treatment X Session interaction (all p’s > 0.05). n = 7 per group.

First, we tested whether nicotine treatment during adolescence would by itself affect operant alcohol self-administration during adulthood. Rats were treated with 5 injections of nicotine (0.4 mg/kg, i.p., every 3^rd^ day) or saline in the home cage during late adolescence, on postnatal days (PND) 45–59 (see Experimental Design). This regimen was previously reported to sensitize rats to nicotine’s psychomotor and dopamine-activating effects^29^, and to form a nicotine-context association that affects drug intake^21^. Next, rats were trained to self-administer alcohol on PND 63–97 (Fig. 1, timeline). To this end, rats were trained in the intermittent access operant self-administration procedure^30^, which consisted of two phases: three weeks of 14-h overnight sessions, employed to facilitate the acquisition of operant alcohol self-administration^30, 31^, followed by two weeks of 60-min sessions. All self-administration sessions were conducted 3 days a week (Sunday, Tuesday and Thursday).

No differences were found between nicotine- and saline-pre-treated rats in active lever presses (Fig. 1a and b) and in alcohol intake (Fig. 1c and d) during adulthood, in the overnight or the 60-min sessions. Moreover, there were no differences between the groups in the number of lever presses and alcohol intake during the first hour of the overnight sessions (Supplemental Figure S1). No significant effects were found for number of inactive lever presses (p > 0.05). These results suggest that nicotine pre-treatment on its own during adolescence does not affect subsequent alcohol intake in adulthood.

#### Experiment 2

**Figure 2:**
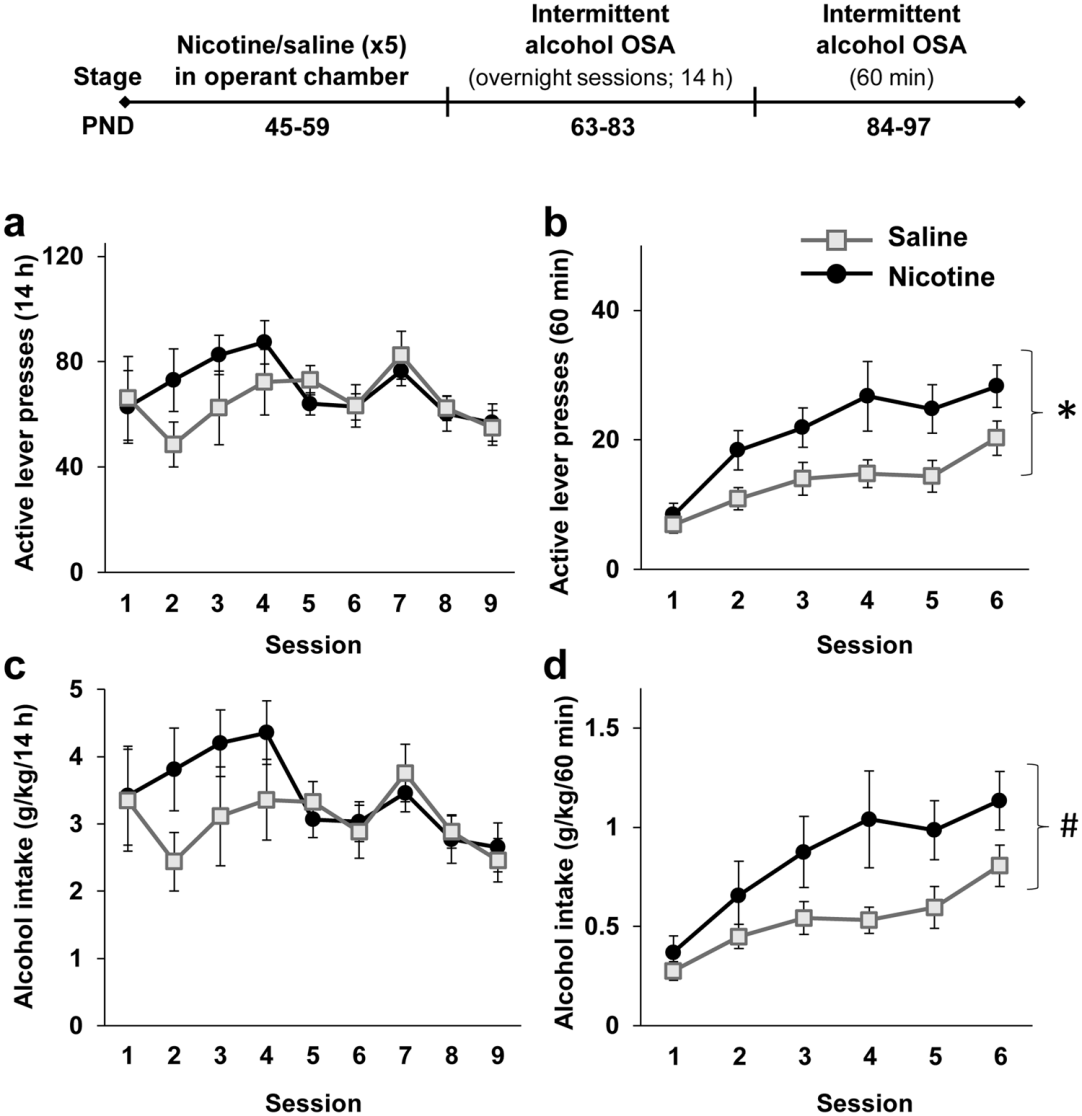
Adolescent nicotine pre-treatment in the operant chambers enhances operant alcohol self-administration in adulthood. Rats were treated with 5 nicotine or saline injections on PND 45–59, and were confined for 2 h to the operant self-administration (OSA) chambers. On PND 63 rats began intermittent overnight operant alcohol self-administration training three days a week (fixed ratio 1; FR1) for 3 weeks. On PND 84, rats began intermittent operant training (FR1) of 60 min, three days a week for 2 weeks. (**a–d**) Means ± SEM of the number of active lever presses (**a** and **b**) or alcohol intake normalized to body weight (**c** and **d**) during the overnight (**a** and **c**) or 60-min (**b** and **d**) sessions. Mixed-model ANOVA; Overnight sessions: A main effect of Session for active-lever presses [F(8,112) = 2.90, p < 0.01] and for alcohol intake [F(8,112) = 3.13, p < 0.005], but no significant main effect of Pre-treatment and no significant Pre-treatment X Session interaction (p > 0.05); 60-min sessions: A main effect of Pre-treatment for active lever presses [F(1,14) = 5.61, p < 0.05] and marginally significant for alcohol intake [F(1,14) = 4.54, p = 0.051], a main effect of Session for active lever presses [F(5,70) = 17.64, p < 0.0001] and for alcohol intake [F(5,70) = 13.66, p < 0.0001], but no significant Pre-treatment X Session interaction (p > 0.05). n = 8 per group; *p < 0.05; ^#^p < 0.06.

Since we found that alcohol consumption in adulthood is not affected by pre-treatment with nicotine *per se*, we next tested whether contextual cues associated with nicotine would affect alcohol intake. Rats were administered with nicotine or saline during adolescence (5 injections on PND 45–59) and immediately following each injection, were placed in the operant chambers, to form a context-nicotine association. On PND 63, rats began intermittent access operant self-administration training (Fig. 2, timeline). We found that rats that were pre-treated with nicotine showed higher levels of operant response and alcohol intake in the 60-min (PND 84–97), but not in the overnight sessions (PND 63–83), compared to saline-treated rats (Fig. 2). No difference was seen between the groups in the number of lever presses and alcohol intake during the first hour of the overnight sessions (Supplemental Figure S2), and no effects were found for number of inactive lever presses (p > 0.05). These results suggest that consumption of alcohol in adulthood is augmented when nicotine pre-treatment during adolescence is given in the operant self-administration chambers.

#### Experiment 3

Next, we determined whether exposure to nicotine in adolescence, paired or unpaired with the (to be) alcohol self-administration context, would affect alcohol consumption in adulthood. For this end, both groups received nicotine treatment in a similar regimen as in Experiments 1 and 2. However, for one group of rats, nicotine administration was paired with the operant chamber and saline treatment was paired with the home cage, whereas for the control group, saline treatment was paired with the operant chamber and nicotine treatment was paired with the home cage (see Experimental Design).

**Figure 3:**
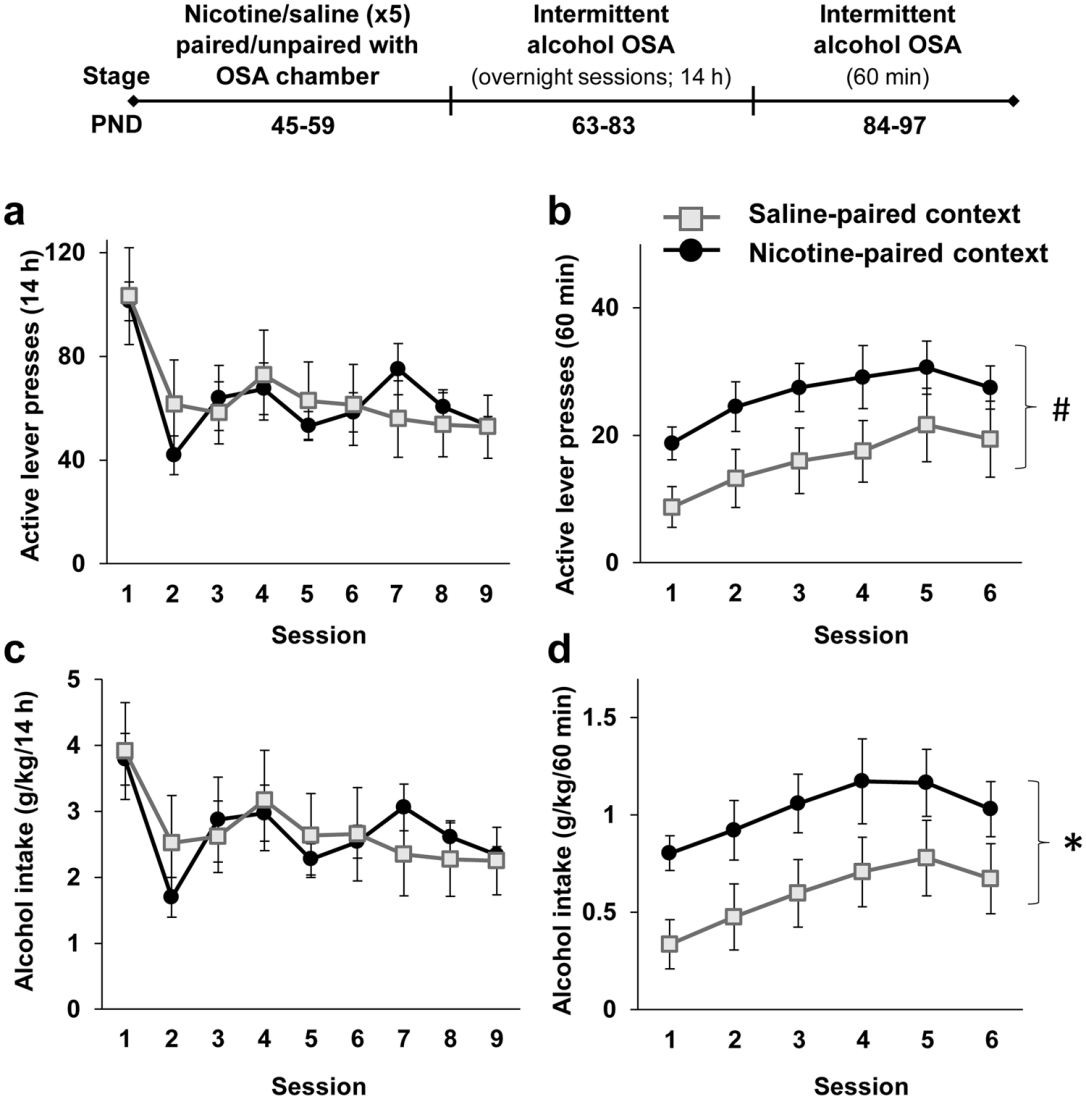
Adolescent nicotine pre-treatment paired with the operant chambers but not with the home cages enhances operant alcohol self-administration in adulthood. Rats were treated with 5 nicotine and saline injections on PND 45–59. Nicotine treatment was either paired with the operant self-administration (OSA) chambers (Nicotine-paired context group), or unpaired with the OSA chamber by placing the rats in the home cage following nicotine treatment, while pairing the chamber with saline (Saline-paired context group). On PND 63 rats began intermittent overnight operant alcohol self-administration training three days a week (fixed ratio 1; FR1) for 3 weeks. On PND 84, rats began intermittent operant training (FR1) of 60 min three days a week for 2 weeks. (**a–d**) Means ± SEM of the number of active lever presses (**a** and **b**) or alcohol intake normalized to body weight (**c** and **d**) during the overnight (**a** and **c**) or 60-min (**b** and **d**) sessions. Mixed-model ANOVA; Overnight sessions: A main effect of Session for active-lever presses [F(8,112) = 9.47, p < 0.0001] and for alcohol intake [F(8,112) = 5.70, p < 0.0001], but no significant main effect of Context-pairing and no significant Context-pairing X Session interaction (p > 0.1); 60-min sessions: A main effect of Context-pairing for alcohol intake [F(1,14) = 4.97, p < 0.05] and marginally significant for active lever presses [F(1,14) = 3.81, p = 0.071], a main effect of Session for active lever presses [F(5,70) = 5.15, p < 0.001] and for alcohol intake [F(5,70) = 4.40, p < 0.005], but no significant Context-pairing X Session interaction (p > 0.1). n = 8 per group; *p < 0.05; ^#^p = 0.071.

We found that when the operant chamber was paired with nicotine (Nicotine-paired context group), rats showed higher levels of operant response for alcohol and enhanced alcohol intake starting on PND 84 (beginning of 60-min training sessions), compared to rats for whom the operant chamber was paired with saline, and unpaired with nicotine (Saline-paired context group; Fig. 3). No difference was seen between the groups in the number of lever presses and alcohol intake during the first hour of the overnight sessions (Supplemental Figure S3), and no effects were found for number of inactive lever presses (p > 0.05). These results indicate that nicotine pre-treatment in adolescence enhances alcohol intake in adulthood only when nicotine treatment has been paired with the operant self-administration context.

### Nicotine-associated contextual memories lead to long-lasting enhancement of alcohol self-administration and relapse

#### Experiment 4

In Experiment 3, nicotine pre-treatment was administered in a familiar environment of the home cage as the control context, whereas the operant chamber was a novel environment for the rats. Thus, to rule out the possibility that the effect of nicotine pre-treatment on alcohol intake was due to novelty of the nicotine-paired operant chamber context, in Experiment 4 we used another, neutral and novel context as the control context. Specifically, both groups received nicotine treatment similar to Experiment 3. However, for the Nicotine-paired context group, nicotine administration was paired with the operant chamber (Context A) and saline treatment was paired with a distinct, novel context (Context B), whereas for the control, Saline-paired context group, saline treatment was paired with the operant chamber and nicotine treatment was paired with context B (see Experimental Design). Moreover, in this experiment we also tested whether the effects of nicotine context would be long lasting, and whether nicotine context would increase alcohol relapse-like behavior after a period of abstinence.

**Figure 4:**
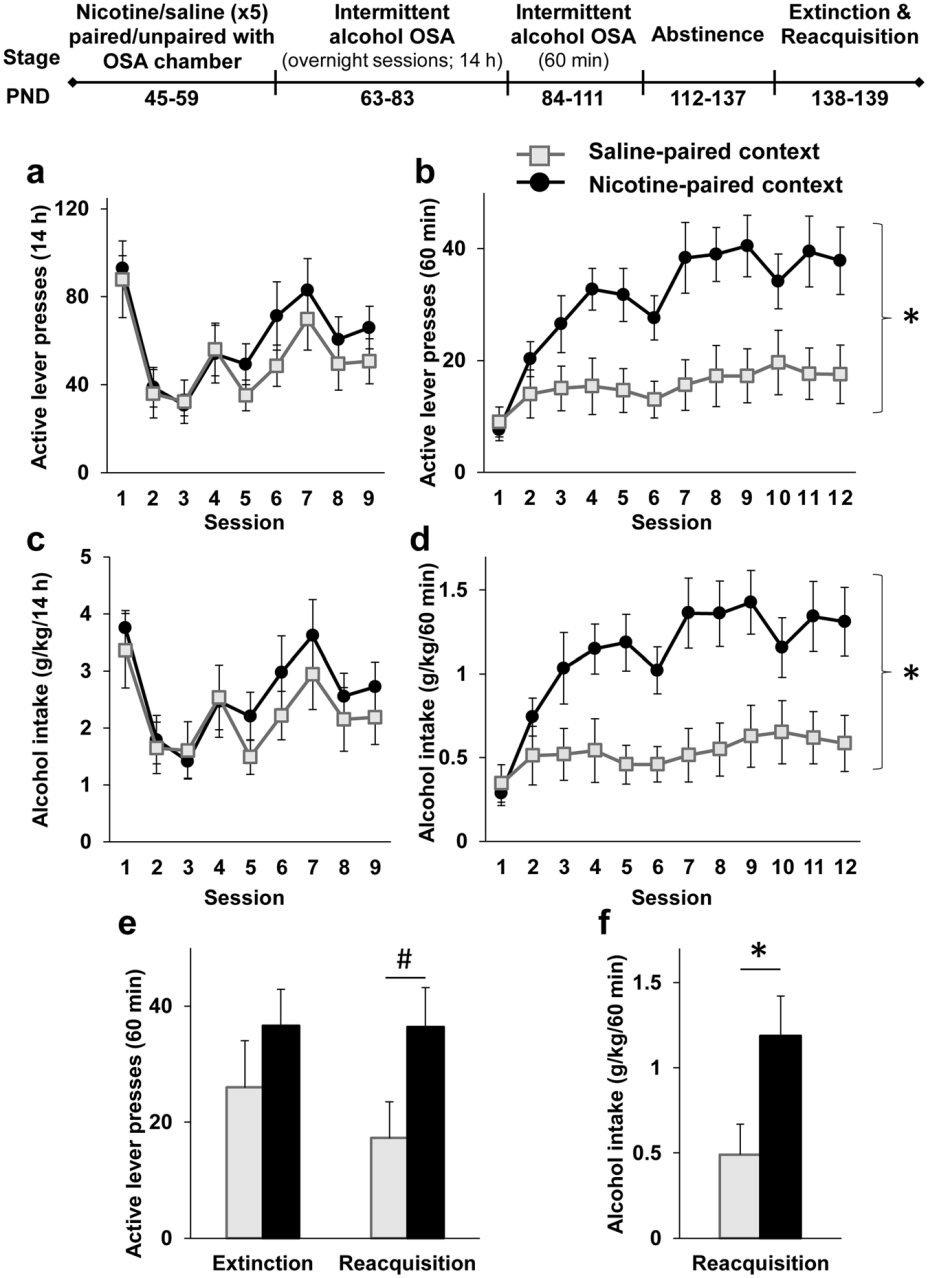
Adolescent nicotine pre-treatment paired with the operant chambers but not with a different context induces long-lasting enhancement of operant alcohol self-administration and relapse in adulthood. Rats were treated with 5 nicotine and saline injections on PND 45–59. Nicotine treatment was either paired with the operant self-administration (OSA) chambers (Nicotine-paired context group), or unpaired with the OSA chamber by placing the rats in a neutral different context following nicotine treatment, while pairing the chamber with saline (Saline-paired context group). On PND 63 rats began intermittent operant alcohol self-administration training three days a week (fixed ratio 1; FR1) for 3 weeks. On PND 84, rats began intermittent operant training (FR1) of 60 min three days a week for 4 weeks. Rats were then subjected to 4 weeks of abstinence in the home cage, followed by an extinction and reacquisition sessions to test relapse to alcohol seeking and drinking, respectively. (**a–f**) Means ± SEM of the number of active lever presses (**a**,**b**,**e**) or alcohol intake normalized to body weight (**c**,**d**,**f**) during the overnight sessions (**a** and **c**), 60-min sessions (**b** and **d**) or extinction and reacquisition sessions (**e**,**f**). Mixed-model ANOVA; Overnight sessions: A main effect of Session for active lever presses [F(8,112) = 8.69, p < 0.0001] and for alcohol intake [F(8,112) = 6.71, p < 0.0001], but no significant main effect of Context-pairing and no significant Context-pairing X Session interaction (p > 0.1); 60-min sessions: A main effect of Context-pairing for active lever presses [F(1,14) = 8.12, p < 0.05] and for alcohol intake [F(1,14) = 8.6, p < 0.05], a main effect of Session for active lever presses [F(11,154) = 9.8, p < 0.0001] and for alcohol intake [F(11,154) = 8.62, p < 0.0001], and a significant Context-pairing X Session interaction for active lever presses [F(11,154) = 3.67, p < 0.001] and for alcohol intake [F(11,154) = 3.95, p < 0.0001], which was driven by simple effects of Session for Nicotine-paired context group (F(11,77) > 9.62, p’s < 0.0001) but not for the Saline-paired context group (p’s > 0.1). Extinction test: active lever presses, t(13) = 1.06, p > 0.1; Reacquisition test: active lever presses, t(13) = 2.06, p = 0.06, alcohol intake, t(13) = 2.31, p < 0.05. n = 7–8 per group; *p < 0.05; ^#^p = 0.06.

Similar to Experiment 3, we found that pairing the operant chambers with nicotine led to increased operant alcohol self-administration compared to pairing the chambers with saline, and this effect emerged only in the 60-min training sessions. Importantly, this enhancement in alcohol self-administration induced by nicotine-associated context lasted 4 weeks (12 sessions) (Fig. 4). No effects were found for number of inactive lever presses (p > 0.05), and no difference was seen between groups trained in this design in the number of lever presses and alcohol intake during the first hour of the overnight sessions (Supplemental Figure S4).

Next, in order to test the long-lasting effects of nicotine-associated context on relapse, rats were given a 4-weeks alcohol abstinence period. Then, we assessed relapse to alcohol seeking by testing alcohol prime-induced operant behavior under extinction conditions (lever presses were not reinforced). In addition, 24 h later we assessed relapse to alcohol drinking in an alcohol prime-induced reacquisition test (lever presses were reinforced).

While we found no significant difference between groups in the extinction session, we observed increased reacquisition of operant alcohol self-administration in the group for which the operant chamber was paired with nicotine, compared to controls (Fig. 4e and f). Interestingly, we found that during the reacquisition session, control rats terminated their lever-pressing after 25–30 min. However, rats from the nicotine-paired context group pressed the lever more than controls from the beginning of the session, and continued to self-administer alcohol throughout the whole 60-min session (Supplemental Figure S5). These results indicate that the enhancing effects of nicotine-associated context on alcohol self-administration are very long-lasting, and affect relapse to alcohol-drinking behavior even after a month of alcohol abstinence, and 3 months after nicotine cessation.

Taken together, Experiments 1–4 indicate that while nicotine pre-treatment on its own has no effect on subsequent alcohol intake, the consumption of alcohol in adulthood is increased if the self-administration context has been paired with nicotine during adolescence. Moreover, we show that this enhancement in alcohol intake is long-lasting, and persists even after 4 weeks of abstinence by increasing relapse to alcohol drinking. Thus, these results suggest that nicotine-associated contextual memories from adolescence convey the enhancement of alcohol consumption and relapse in adulthood.

### Adolescent nicotine pre-treatment does not affect operant self-administration of sucrose in the nicotine-associated context

#### Experiment 5

**Figure 5:**
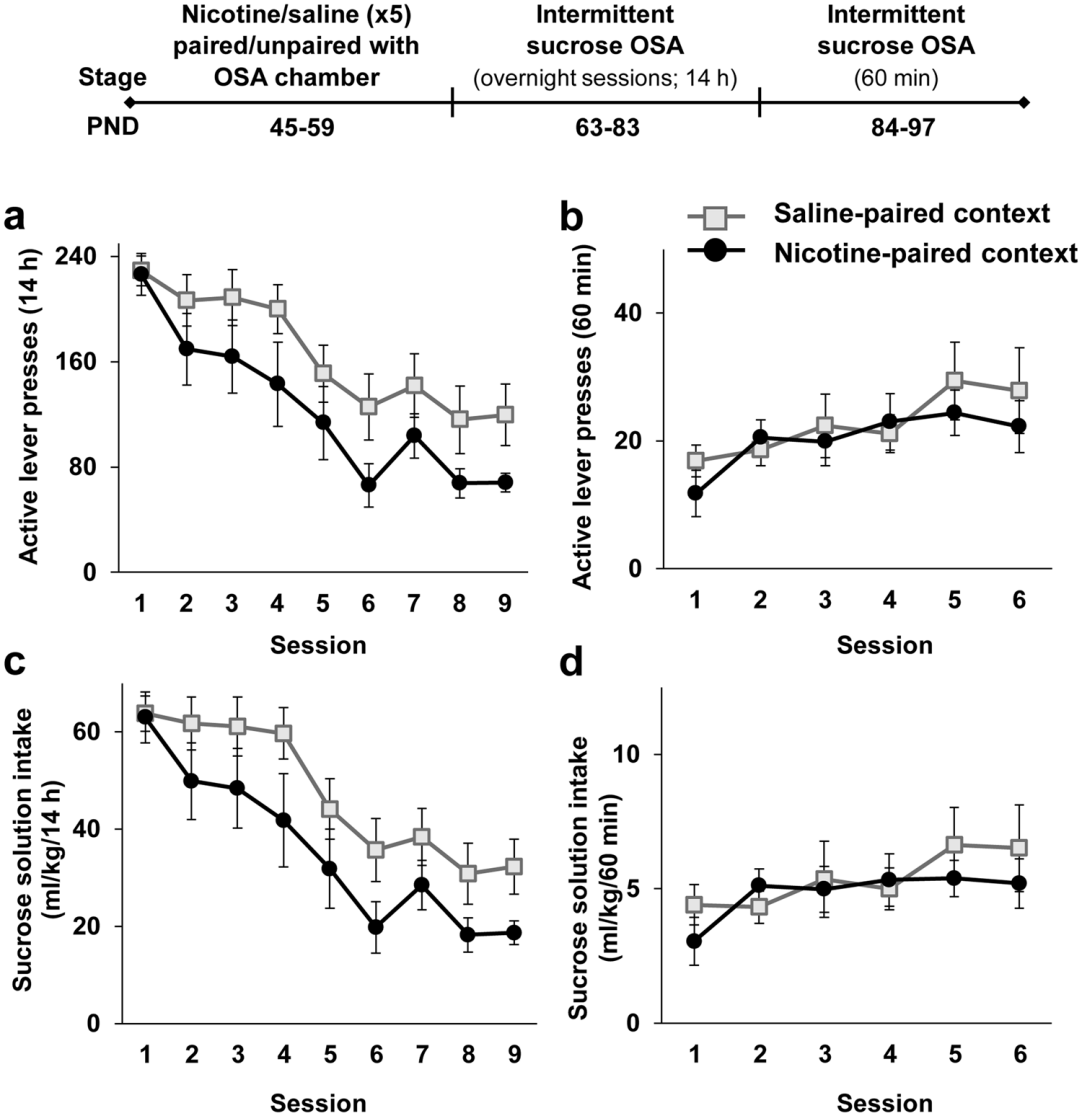
Adolescent nicotine pre-treatment paired with the operant chambers does not affect operant sucrose self-administration in adulthood. Rats were treated with 5 nicotine and saline injections on PND 45–59. Nicotine treatment was either paired with the operant self-administration (OSA) chambers (Nicotine-paired context group), or unpaired with the OSA chamber by placing the rats in a neutral different context following nicotine treatment, while pairing the chamber with saline (Saline-paired context group). On PND 63 rats began intermittent operant self-administration of sucrose solution (6% in the first session, then gradually reduced to 0.5% in the 6^th^ session). Training was conducted three days a week (fixed ratio 1; FR1) for 3 weeks. On PND 84, rats began intermittent operant training (FR1) of 60 min three days a week for 2 weeks. (**a–d**) Means ± SEM of the number of active lever presses (**a** and **b**) or sucrose solution intake normalized to body weight (ml/kg) (**c** and **d**) during the overnight (**a** and **c**) or 60-min (**b** and **d**) sessions. Mixed-model ANOVA; Overnight sessions: A main effect of Session for active-lever presses [F(8,112) = 25.86, p < 0.0001] and for sucrose solution intake [F(8,112) = 28.92, p < 0.0001], but no significant main effect of Context-pairing or Context-pairing X Session interaction (p > 0.1); 60-min sessions: A main effect of Session for active-lever presses [F(5,70) = 4.23, p < 0.01] and for sucrose solution intake [F(5,70) = 2.41, p < 0.05], but no significant main effect of Context-pairing, and no Context-pairing X Session interaction (p’s > 0.1). n = 8 per group.

Next, we tested whether the effects of nicotine-associated context are specific to alcohol-drinking behavior, or can be generalized to natural reinforcers. Rats were given the nicotine treatment and self-administration training as described in Experiment 4, except that lever-press response was reinforced with sucrose solution instead of alcohol. As depicted in Fig. 5, no differences were found between groups in the number of lever presses (Fig. 5a and b) and in sucrose solution intake (Fig. 5c and d), during the overnight and the 60-min sessions. No effects were found for number of inactive lever presses (p > 0.05). These results indicate that the effect of nicotine-associated context is specific to alcohol, and is not generalized to natural reinforcers.

### Re-exposure to nicotine-associated context does not evoke anxiety-like behavior

#### Experiment 6

It is well-established that nicotine can evoke anxiogenic effects under certain conditions^32^, and that alcohol intake is increased by anxiety^33, 34^. Therefore, we next tested whether the increased alcohol intake we observed could be attributed to anxiety/stress induced by the re-exposure to the nicotine-associated context. To this end, we treated rats with nicotine in the same pairing/unpairing protocol as in Experiment 4 (see Experimental design). Thus, the operant chamber was associated with nicotine for one group (Nicotine-paired context group), and with saline for the other group (control, Saline-paired context group), whereas context B was paired with saline and nicotine, respectively. On PND 84, the age in which the effect of nicotine-context on alcohol intake emerged, we tested whether a short re-exposure to the operant chamber (applying nicotine context re-exposure for one group and saline context re-exposure for the control group) induces anxiety-like behaviors in the Nicotine-paired context group, as measured by a subsequent elevated plus maze (EPM) test, conducted outside of the nicotine-associated context.

**Figure 6:**
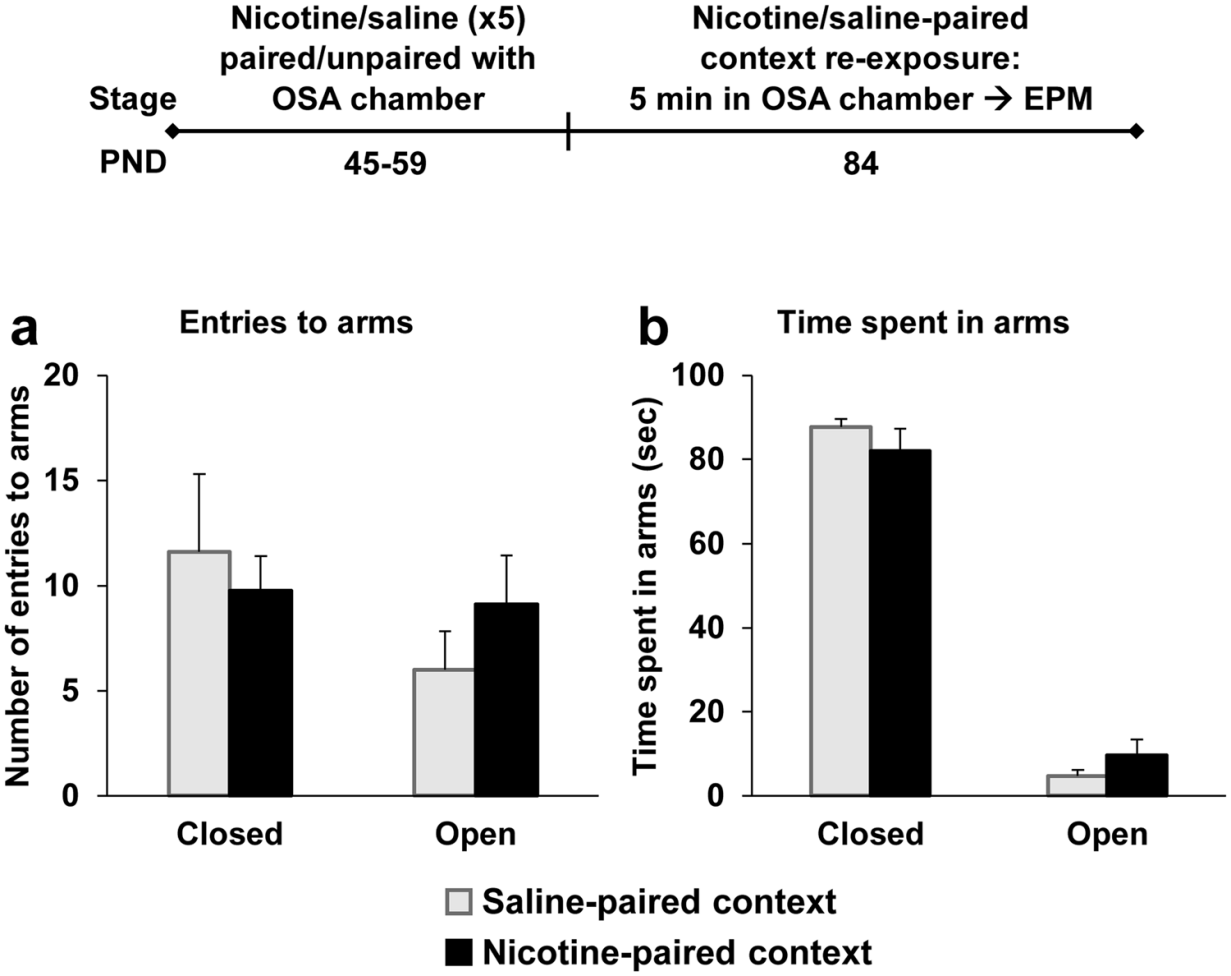
Re-exposure to nicotine-associated context does not evoke anxiety-like behavior. Rats were treated with 5 nicotine and saline injections on PND 45–59 either in the operant self-administration (OSA) chambers or in a different context. The OSA chambers were paired with nicotine treatment for the Nicotine-paired context group (rats received saline in the different context), or with saline for the control, Saline-paired context group (rats received nicotine in the different context). On PND 84, anxiety-like behavior was assessed by elevated plus maze (EPM) after a 5-min exposure to the OSA chambers (applying a re-exposure to the nicotine-associated context only for the Nicotine-paired context group, and re-exposure to the saline-paired context for the control group). (**a**) Entrance frequency to the closed or open arms of the maze. (**b**) Time spent in closed or the open arms. Bar graphs represent means ± SEM. All t(14) < 1.25, p’s > 0.05. n = 8 per group.

All rats were placed for 5 min in the operant chamber (which was associated with nicotine only for the Nicotine-paired context group), and then underwent the EPM test. We found no differences between the groups in time spent in the open or closed arms, or in arm-entrance frequency (Fig. 6), indicating that re-exposure to the nicotine context did not increase anxiety, tested thirty minutes after exposure to this context.

### Re-exposure to nicotine-associated context decreases *Gdnf* expression in the ventral tegmental area (VTA)

We have recently shown that a knockdown of *Gdnf* expression in the VTA by shRNA leads to a sharp increase in alcohol self-administration, whereas overexpressing the growth factor decreases alcohol consumption^27, 28^. Therefore, in the last set of experiments we tested whether re-exposure to the nicotine-associated context, which led to increased alcohol intake (Figs 2–4), would alter the mRNA expression of *Gdnf*. We focused on the VTA and NAc, which are the brain regions where *Gdnf* was shown to regulate alcohol intake^27, 28, 35, 36^.

#### Experiments 7–8

We trained rats in the nicotine-context pairing/unpairing protocol as described above (Experiment 4, see Experimental Design). Thus, all rats were exposed to the same amount of nicotine, which was associated with different contexts; the operant chamber was associated with nicotine for one group, and with saline for the other group. Two weeks (PND 73, Experiment 7) or a month (PND 90, Experiment 8) after the completion of the nicotine-pre-treatment regimen, rats were re-exposed to the operant chambers (applying nicotine context re-exposure for one group and saline context re-exposure for the control group). Rats did not receive operant alcohol self-administration prior to the re-exposure to the operant chambers. Rats were sacrificed 30 min after a 5-min context re-exposure, and *Gdnf* mRNA expression was assessed by qRT-PCR.

**Figure 7:**
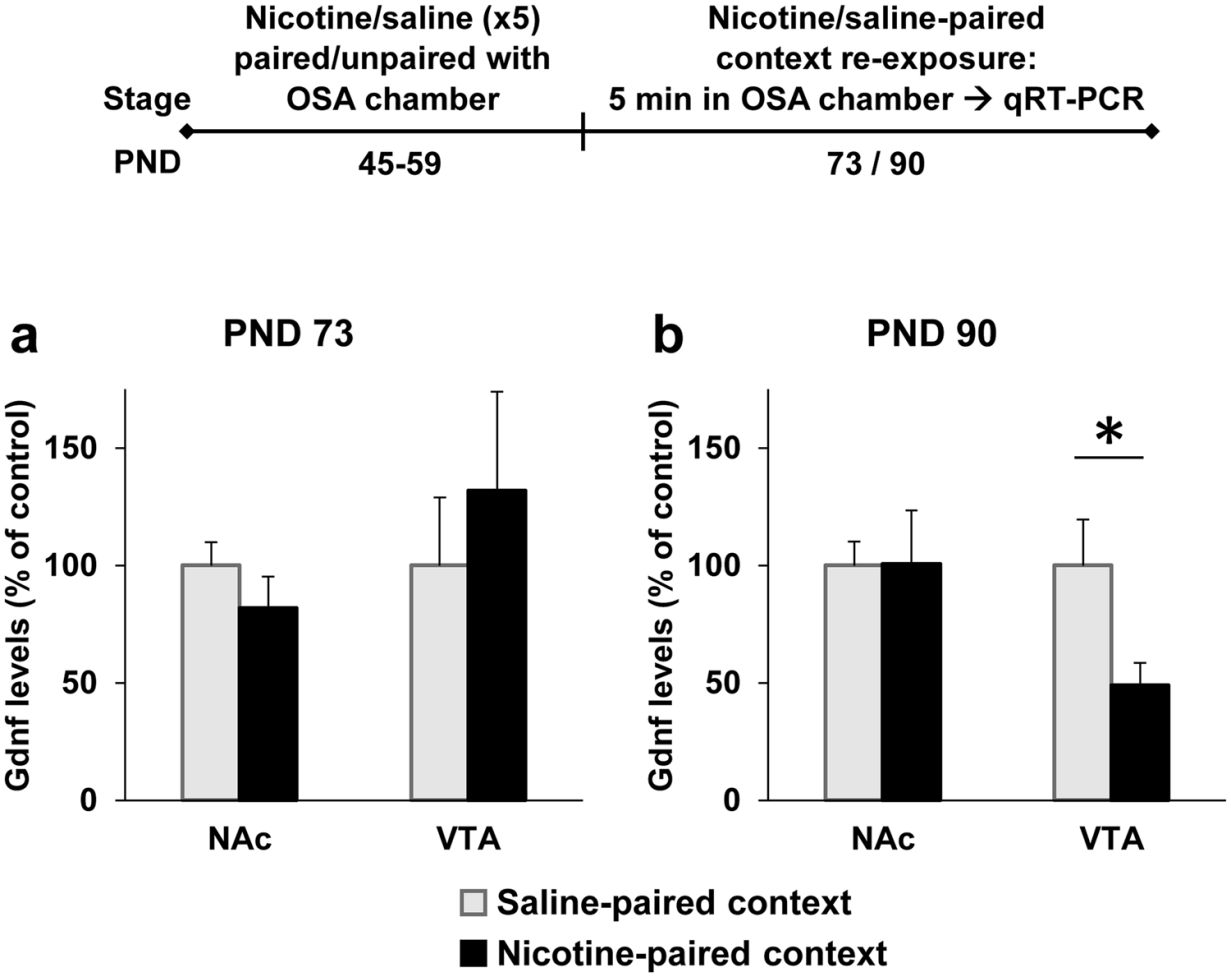
Re-exposure to nicotine-associated context a month after nicotine pre-treatment reduces *Gdnf* expression in ventral tegmental area (VTA). Rats were treated with 5 nicotine and saline injections on PND 45–59 either in the operant self-administration (OSA) chambers or in a different context. The OSA chambers were paired with nicotine treatment for the Nicotine-paired context group (received saline in a different context), or with saline for the control, Saline-paired context group (received nicotine in a different context). Brains were dissected 30 min after a 5-min re-exposure to the OSA chambers (applying a re-exposure to the nicotine-associated context only for the Nicotine-paired context group, and re-exposure to the saline-paired context for the control group) at the age of PND 73 (**a**) or PND 90 (**b**). *Gdnf* mRNA expression levels in NAc and VTA were determined by qRT-PCR and normalized to *Gapdh*. Bar graphs represent means ± SEM. VTA at PND 90: t(11) = 2.47, p < 0.05. All other p’s > 0.05. n = 5–7 per group; *p < 0.05.

We found that the expression of *Gdnf* did not differ between the group that was re-exposed to the Nicotine-paired context and the control, Saline-paired context group in the NAc or the VTA, when re-exposure was conducted 2 weeks after the completion of the nicotine pre-treatment regimen (Fig. 7a). However, when context re-exposure was conducted one month after nicotine treatment, we found that the Nicotine-paired context group displayed 50% reduction in *Gdnf* expression in the VTA, compared to the control, Saline-paired context group (Fig. 7b). No effect was found in the NAc.

Taken together, these findings suggest that re-exposure to the nicotine-associated context leads to a decrease in the expression of *Gdnf* in the VTA, a reduction that enhances alcohol consumption and relapse, as we previously demonstrated^27^.

## Discussion

We show here that operant alcohol self-administration in adult rats is increased, when the environmental context of the consumption has been associated with nicotine during late adolescence. Importantly, it is not nicotine pre-treatment *per se* that affects subsequent alcohol self-administration, as the effect emerges only when nicotine treatment has been paired with the operant chamber. Moreover, this effect is seen even long-after the cessation of nicotine treatment (3 months), and after a period of alcohol abstinence. In addition, we show that a short re-exposure to nicotine-associated context leads to reduction in the mRNA expression of *Gdnf* in the VTA, a growth factor which was previously shown to gate alcohol intake^27^. Thus, we suggest that the mere retrieval of nicotine-associated contextual memories leads to increased alcohol consumption in adulthood, possibly through *Gdnf* downregulation.

Our results indicate that a history of nicotine exposure on its own is not predictive of enhanced alcohol intake. While several studies have shown that nicotine treatment acutely increases alcohol intake (e.g., refs 11, 12, 16 and 17) and alcohol relapse^12, 17^, only a few studies tested the effects of a repeated nicotine treatment on subsequent alcohol self-administration (e.g., refs 10 and 37). Similar to our results, Kemppainen *et al*.^37^ found no effect of adolescent nicotine pre-exposure on subsequent alcohol intake in adulthood. However, a challenge of nicotine given to rats with a history of nicotine exposure increased alcohol self-administration compared to nicotine-naïve controls^37^, suggesting that nicotine pre-treatment leads to sensitizing effects relevant to alcohol intake. In contrast, Blomqvist *et al*.^10^ found that subchronic nicotine treatment increased subsequent alcohol intake. However, in that study, rats were first trained to self-administer alcohol, and only then were treated with nicotine during alcohol withdrawal. Therefore, it is possible that in adult rats with a history of alcohol intake, nicotine given during alcohol abstinence enhances the alcohol deprivation effect^38^, or the incubation of alcohol craving^39^.

We found that the shared context for nicotine pre-treatment and alcohol consumption was required to induce an increase in operant alcohol self-administration. Interestingly, nicotine-associated context has also been shown to increase the operant self-administration of amphetamine^21^ and of nicotine itself^23^. Together, these results imply that nicotine pre-treatment can enhance the incentive salience of a context even long after the context-nicotine pairing, in a way that will stimulate the subsequent consumption of other drugs, which are considered more harmful to the user and/or to society^40^.

Interestingly, these context-specific cross-drug effects are reminiscent of the context-specific psychomotor sensitization effect, i.e., psychomotor sensitization selectively expressed in the drug-associated environmental context, but not in another environment^41, 42^. Moreover, context-specific sensitization can be transferred across drugs^43^, suggesting that the effect on alcohol self-administration we observed is possibly due to the context-specific sensitizing effect of nicotine. Importantly, this effect is unlikely to derive from nicotine-related stress, as we show here that the nicotine-paired context did not elicit anxiety-like behavior, when tested immediately following re-exposure to this context. Finally, nicotine-associated context had no effects on operant self-administration of sucrose solution, suggesting that enhancing effect of nicotine context on alcohol/drug consumption is not generalized to natural reinforcers

The increase in alcohol self-administration in the nicotine-paired context emerged only approximately three weeks after the cessation of nicotine treatment. While the nature of this delayed-onset effect is not clear, it is possible that it involves an “incubation” period, and is therefore only manifested following this period. Indeed, drug sensitization, like drug craving^39^, was shown to undergo an “incubation” period leading to increased sensitization over time^44, 45^. Thus, it is possible that nicotine sensitization is incubating and gradually enhancing during the first few weeks after the termination of nicotine treatment, and is therefore emerging behaviorally in a delayed manner.

Importantly, the effect of nicotine context on drinking emerged concurrently with the change from an overnight to a 60-min session training. However, the timing, rather than the session length, seems to be a critical factor in the emergence of the effect, as we found no difference in operant behavior and alcohol intake during the first hour of the 14-h sessions. Nevertheless, it is possible that at least in part, training in shorter sessions during daytime contributed to the late emergence of nicotine context effect on alcohol intake.

Interestingly, we found that the emergence of the effects of nicotine context exposure on alcohol self-administration is temporally-correlated with its effect on *Gdnf* expression in the VTA. Thus, *Gdnf* expression was not altered by re-exposure to the nicotine context two weeks after the termination of the nicotine treatment regimen. Rather, the reduction in *Gdnf* expression was found when the context re-exposure was conducted a month after nicotine cessation, in a timing parallel to the late-onset effect on alcohol self-administration. It is possible that the delayed effect on *Gdnf* expression in the VTA also reflects an “incubation” period, and that only when GDNF levels are reduced in response to nicotine context exposure, alcohol intake is increased.

Relevantly, Tobacco smoking was recently reported to be associated with genetic variants in the *Gdnf* gene^46^, and the synergistic effects of nicotine and alcohol were previously shown to be localized to the VTA, and to involve a reduction in mesolimbic *Gdnf* expression^26^. Moreover, we recently found that downregulation of *Gdnf* in the VTA leads to increased alcohol intake and relapse^27, 28^. Taken together, these findings raise the possibility that the reduced *Gdnf* expression in the VTA induced by the re-exposure to nicotine context is a relevant mechanism for the increase in alcohol consumption observed in rats self-administering alcohol in the nicotine-associated context. It should be noted, however, that our findings here only provide a temporal correlation, and that future studies should address the causality of the suggested *Gdnf* mechanism.

Nicotine has been widely described as a primary ‘gateway drug’ in the ‘gateway hypothesis’, which postulates that tobacco or alcohol use precedes and promotes the abuse of various illicit drugs, and is mostly based on epidemiological studies^47, 48^. Can nicotine also gate alcohol use? In the U.S., the mean age of onset for cigarette smoking in 2012 was 15.6 years, and 16.5 years for alcohol use^48^, suggesting that in average, tobacco use precedes alcohol use. Moreover, tobacco use is a strong predictor of alcohol abuse^49^, and early-onset tobacco smokers are more likely to abuse alcohol^5^. Importantly, our findings raise the possibility that the environment, and possibly other cues associated with cigarette smoking among adolescents, might function as potent enhancers of alcohol consumption later in life, even after smoking cessation.

To conclude, our findings imply that nicotine-associated contextual memories are a crucial element mediating the effects of nicotine pre-exposure during adolescence on alcohol consumption in adulthood. The fact that these contextual memories are capable of affecting alcohol intake even a long-time after the context-nicotine pairing, suggests that nicotine produces a strong, long-lasting and persistent enhancement of the incentive salience of the context it had been associated with, during adolescence. In adulthood, this context becomes a powerful enhancer of alcohol intake.

## Materials and Methods

### Drugs and reagents

Fast SYBR® Green Master Mix (#4385617), TRIzol reagent (#13150101), and RevertAid reverse transcription kit (#0442) were purchased from Thermo-Fisher Scientific (San Jose, CA, USA). All DNA oligonucleotides, and nicotine as (−)-nicotine hydrogen tartrate (N5260), were obtained from Sigma-Aldrich (Rehovot, Israel). Ethyl alcohol (absolute) was purchased from Gadot (Haifa, Israel).

### Animals

Male Long–Evans rats (Tel Aviv University), weighing 140–200 g at the beginning of experiment, were housed under a 12 h-light/dark cycle (lights on at 7:00 a.m.) with food and water available *ad libitum*. All experimental protocols were approved by, and conformed to, the guidelines of the Institutional Animal Care and Use Committee of Tel Aviv University, and to the guidelines of the NIH (animal welfare assurance number A5010-01). All efforts were made to minimize the number of animals used.

### Intermittent 20% alcohol operant self-administration

This procedure was previously described by Simms *et al*.^30^ (also see Carnicella, *et al*.^31^). Briefly, for the first 3 weeks, rats were placed three times a week (Sunday, Tuesday, and Thursday; total 9 sessions) in operant self-administration chambers (Med Associates, Georgia, VT) for a 14-h overnight session on a fixed ratio 1 (FR1) schedule of reinforcement (0.1 ml 20% alcohol solution (v/v) following a single active lever press). Rats were then trained in 60-min FR1 sessions three times a week for 2 additional weeks (6 sessions) in Experiment 1–3, and for 4 additional weeks in Experiment 4. Number of lever presses on each lever (active and inactive), as well as the number of reward deliveries, were recorded.

### Post-abstinence extinction and reacquisition tests

To test relapse to alcohol seeking and drinking after a 4-week alcohol abstinence period, we used an extinction session and a reacquisition test, respectively, as we previously described^50^.

#### Extinction

Relapse to alcohol seeking was assessed by testing operant behavior under extinction conditions in a single test session. Thus, rats were placed in the operant chambers for a 60-min session similarly to the self-administration training sessions, except that no alcohol was delivered following either lever presses. In addition, an alcohol prime (20%, 0.2 ml) was non-contingently delivered at the beginning of the session.

#### Reacquisition

Relapse to alcohol consumption was assessed in a reacquisition session, taking place 24 h after the extinction session. This session was identical to the extinction test stage, except that alcohol was delivered following lever presses (at FR1) as in the previous operant self-administration sessions.

### Intermittent Sucrose operant self-administration

Operant sucrose self-administration procedure was similar to the alcohol self-administration procedure. Each active lever-press led to a delivery of 0.1 ml of sucrose solution. Sucrose concentration was gradually decreased, from 6% sucrose (w/v) in the first overnight session to 0.5% in the 6^th^ overnight session. The latter concentration remained stable for the following overnight and 60-min self-administration sessions.

### Elevated Plus Maze (EPM)

For anxiety measurement, animals were tested in an elevated plus maze (EPM) test, as previously described^51, 52^. The apparatus was made of acrylic glass, and consisted of four 50 × 10-cm arms: two open arms with 3-mm high margins and two enclosed arms by 30-cm high walls. Each arm of the maze was attached to sturdy metal legs elevated 100-cm off the ground. Rats were placed in an open arm, facing the center of the maze. Entries to, and time spent in the open and closed arms, were recorded by a video-tracking system (EthoVision XT 11.5, Noldus, Wageningen, the Netherlands) for 5-min.

### Quantitative reverse transcriptase polymerase chain reaction (qRT-PCR)

Selected brain regions were dissected from fresh brain tissues. RNA was extracted with TRIzol reagent and precipitated with 100% ethanol and 0.3 M NaAcetate. mRNA was reverse transcribed with RevertAid cDNA synthesis kit. Expression was quantified via quantitative real time PCR (StepOnePlus; Applied Biosystems, Foster City, CA, USA) using the ΔΔCt method. We used the following primers to amplify specific cDNA regions: *Gdnf*, forward 5′-GACGTCATGGATTTTATTCAAGCC-3′; reverse 5′-CCGGTTCCTCTCTCTTCGAG-3′; *Gapdh*, forward 5′-GCAAGAGAGAGGCCCTCAG-3′; reverse 5′-TGTGAGGGAGATGCTCAGTG-3′.

### Experimental Design

#### Experiments 1–2

*Effects of adolescent nicotine pre-treatment in the home cage or in the operant chamber on operant alcohol self-administration in adulthood*. Rats were administered with nicotine (0.4 mg/kg, i.p., dissolved in 0.9% saline and adjusted to pH 7.0 with NaOH) or saline, one injection every third day, a total of five injections starting on PND 45 similar to the doses and regimen previously described by Cortright *et al*.^21^. Following each injection, rats were placed back in their home cage (Experiment 1), or were confined to the operant self-administration chambers for 2 h (Experiment 2). At PND 63, rats started intermittent access operant alcohol self-administration training. We used a mixed-model design with the between-subjects factor of Pre-treatment (nicotine, saline) and the repeated measurements factor of Sessions (9 for the overnight training; 6 for the 60-min training).

#### Experiments 3

*Operant alcohol self-administration in nicotine- vs. saline-paired context in nicotine pre-treated rats*. Nicotine administration was either paired or unpaired with the operant chambers, similar to the regimen previously described by Cortright *et al*.^21^. Specifically, on day 1 (PND 45) rats were administered with nicotine (Nicotine-paired context group) or saline (Saline-paired context group) and were immediately confined to operant chambers for 2 h. On day 2, rats received saline (Nicotine-paired context group) or nicotine (Saline-paired context group) injections and were returned to home cages. No treatment was given on day 3. This 3-day block was repeated five times. Thus, all rats received nicotine, and spent an equal amount of time in the operant chamber prior to the beginning of the intermittent access operant alcohol self-administration training procedure, which started on PND 63. We used a mixed-model design with the between-subjects factor of Context-pairing (Nicotine-paired context, Saline-paired context) and the repeated measurements factor of Sessions (9 for the overnight training; 6 for the 60-min training).

#### Experiments 4

*Operant alcohol self-administration in nicotine- vs. saline-paired context in nicotine pre-treated rats – assessment of long-term effects and relapse*. The design of this experiment was similar to Experiment 3, except for several changes: First, instead of the home cage as a control context, we used a novel environment (Context B). Context B consisted of an empty acrylic glass cage (without food, water or bedding) placed on a cart in a different room with eucalyptus oil odor. Second, to assess the long-lasting effects of nicotine context on alcohol intake, training in the 60-min session lasted 4 weeks (12 sessions). After the 12^th^ self-administration session, the self-administration training was stopped and rats were abstinent from alcohol for a period of 4 weeks. After abstinence, relapse to drug seeking was assessed by measuring the operant behavior under extinction conditions (lever presses were not reinforced). The next day, relapse to alcohol drinking was assessed by a reacquisition test (lever presses were reinforced similarly to the training stage).

#### Experiments 5

*Operant sucrose self-administration in nicotine- vs. saline-paired context in nicotine pre-treated rats*. The design of this experiment was similar to Experiment 4, except that rats were trained to self-administer sucrose solution rather than alcohol. Training in the 60-min session lasted 2 weeks (6 sessions).

#### Experiment 6

*Effects of re-exposure to the nicotine-paired context on anxiety-like behavior*. Rats were injected with nicotine in a pairing/unpairing design, similar to Experiment 4. Thus, all rats received the same number of nicotine injection, paired with the operant chamber, or with context B. On PND 84 (age starting the intermittent 60-min training sessions of operant alcohol self-administration in Experiments 1–4), rats were placed for 5 min in the operant chamber (re-exposure to nicotine context only for the Nicotine-paired context group; and to the saline-paired context for the control group). Thirty min later, rats were tested for anxiety-like behavior in the EPM.

#### Experiments 7–8

*Effects of re-exposure to nicotine-paired context on the expression of Gdnf*Rats were administered with nicotine in a pairing/unpairing design, similar to Experiment 4. Thus, all rats received the same number of nicotine injection, paired with the operant chamber, or with context B. On PND 73 (2 weeks after the completion of the nicotine pre-treatment regimen; Experiment 7), or on PND 90 (a month after the completion of the nicotine pre-treatment regimen; Experiment 8) rats were placed for 5 min in the operant chamber (re-exposure to nicotine context only for the Nicotine-paired context group; and to the saline-paired context for the control group). Thirty min later, rats were sacrificed, and brains tissues were processed for qRT-PCR. Since relative gene expression for each brain region was normalized to the Saline-paired context group of each time point in these separate experiments, we analyzed each time point with unpaired t-test with a factor of Context re-exposure (Nicotine-paired context, Saline-paired context).

### Statistical analysis

Number of alcohol-reward deliveries obtained during the operant alcohol self-administration sessions was used to calculate alcohol consumption levels, normalized to body weight. Lever press (active or inactive lever) and consumption data were analyzed with a mixed-model ANOVA, with a between-subjects factor of treatment group (nicotine vs. saline, or nicotine-paired context vs. saline-paired context), and a repeated measures factor of self-administration sessions. Data of the overnight and 60-min sessions were analyzed separately. Significant interactions were further analyzed using simple effects analysis. Data of the extinction and reacquisition sessions, anxiety indices from the EPM and data generated from qRT-PCR experiments were analyzed using unpaired t-tests. Gene expression levels were normalized to *Gapdh* expression as previously described^27, 35, 53^, and each brain region was normalized to its own Saline-paired context control group.

## Electronic supplementary material

Supplementary Information
