# Supplementary material for: Re-exposure to nicotine-associated context from adolescence enhances alcohol intake in adulthood
Source: Sci Rep. 2017 May 30;7:2479. doi: 10.1038/s41598-017-02177-2 (PMC5449395; doi:10.1038/s41598-017-02177-2)
Supplement: Supplementary file 1 — Supplementary Information [file 41598_2017_2177_MOESM1_ESM.pdf]

Supplementary Figures S1-S5

Re-exposure to nicotine-associated context from adolescence enhances alcohol intake in adulthood

Dor Zipori, Yossi Sadot-Sogrin, Koral Goltseker, Oren Even-Chen, Nofar Rahamim, Ohad Shaham, Segev Barak

School of Psychological Sciences and the Sagol chool of Neuroscience, Tel Aviv University, Tel Aviv, Israel

Figure S1, Zipori et al.

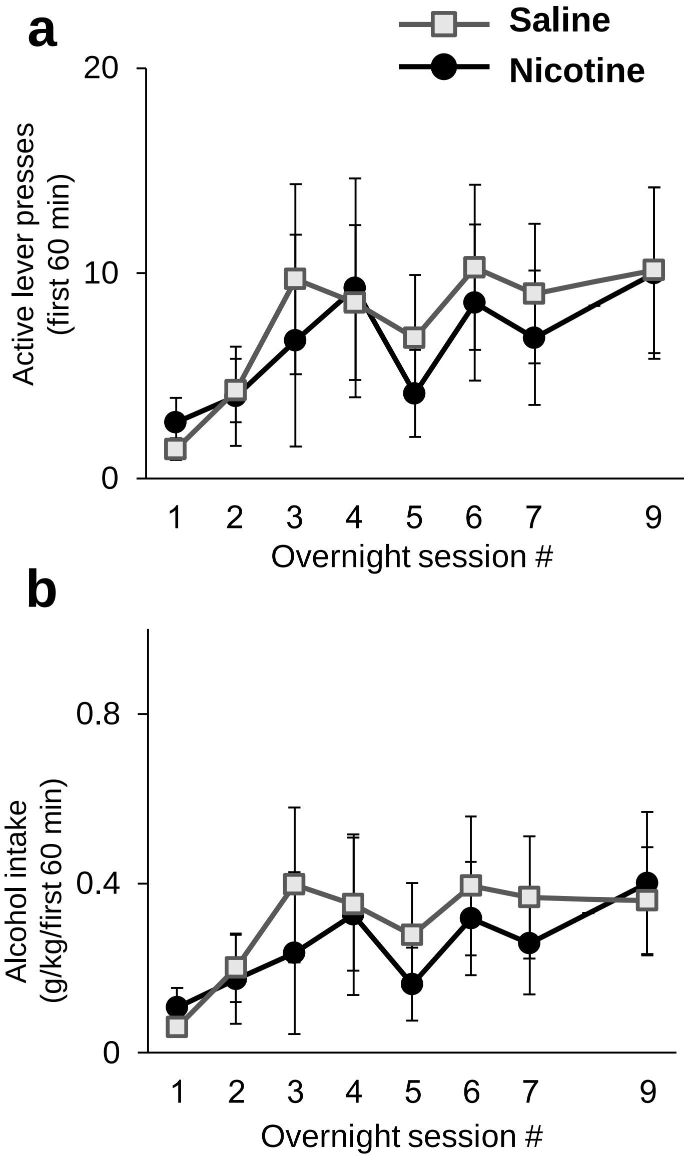

Figure S1. No effects of nicotine pre-treatment in the home cage on operant alcohol self-administration during the first hour of intermittent overnight sessions. a-b. Mean ± SEM number of active lever presses (a) and alcohol intake normalized to body weight (b) during the first 60 min of overnight sessions. Mixed-model ANOVA; A main effect of Session for active-lever presses [$F(7,84)=2.47$, $p<0.05$] and for alcohol intake [$F(7,84)=2.22$, $p<0.05$], but no significant main effect of Pre-treatment and no significant Pre-treatment X Session interaction (all p's>0.05). n=7 per group. Data from session #8 was lost due to a technical failure.

Figure S2, Zipori et al.

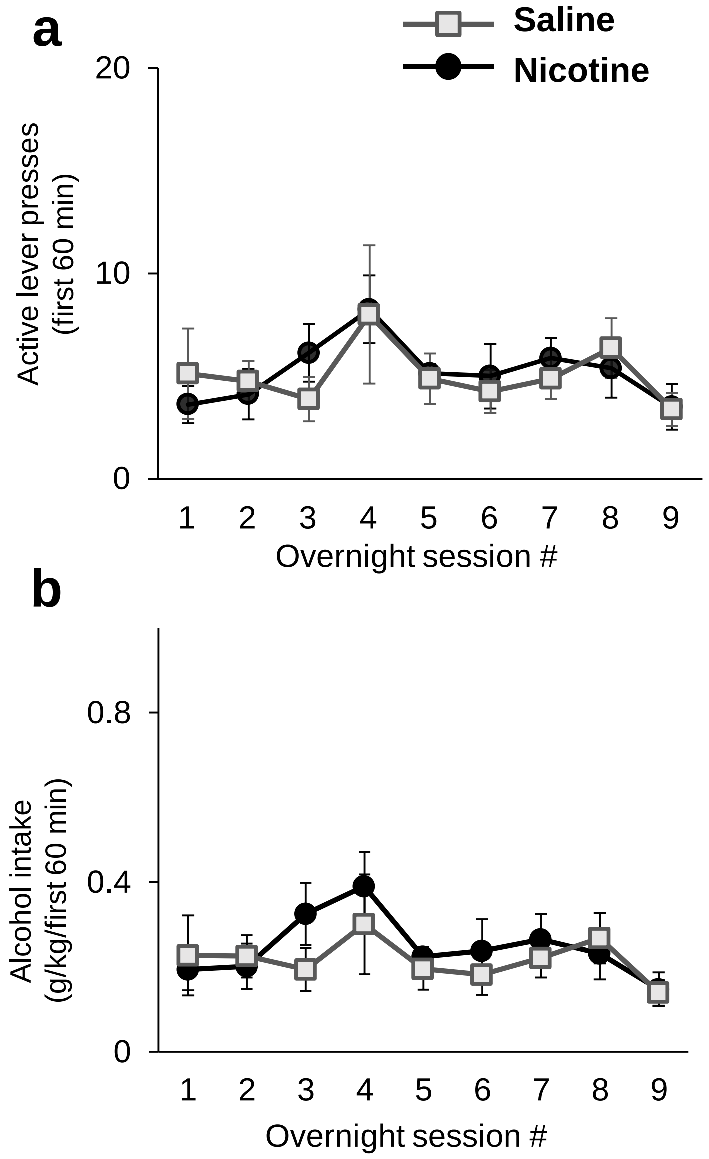

Figure S2. No effects of nicotine pre-treatment in the operant chamber on operant alcohol self-administration during the first hour of intermittent overnight sessions. a-b. Mean ± SEM number of active lever presses (a) and alcohol intake normalized to body weight (b) during the first 60 min of overnight sessions. Mixed-model ANOVA; No significant main effects of Pre-treatment and of Session and no significant Pre-treatment X Session interaction, for both active-lever presses and alcohol intake (all p's>0.05). n=8 per group.

Figure S3, Zipori et al.

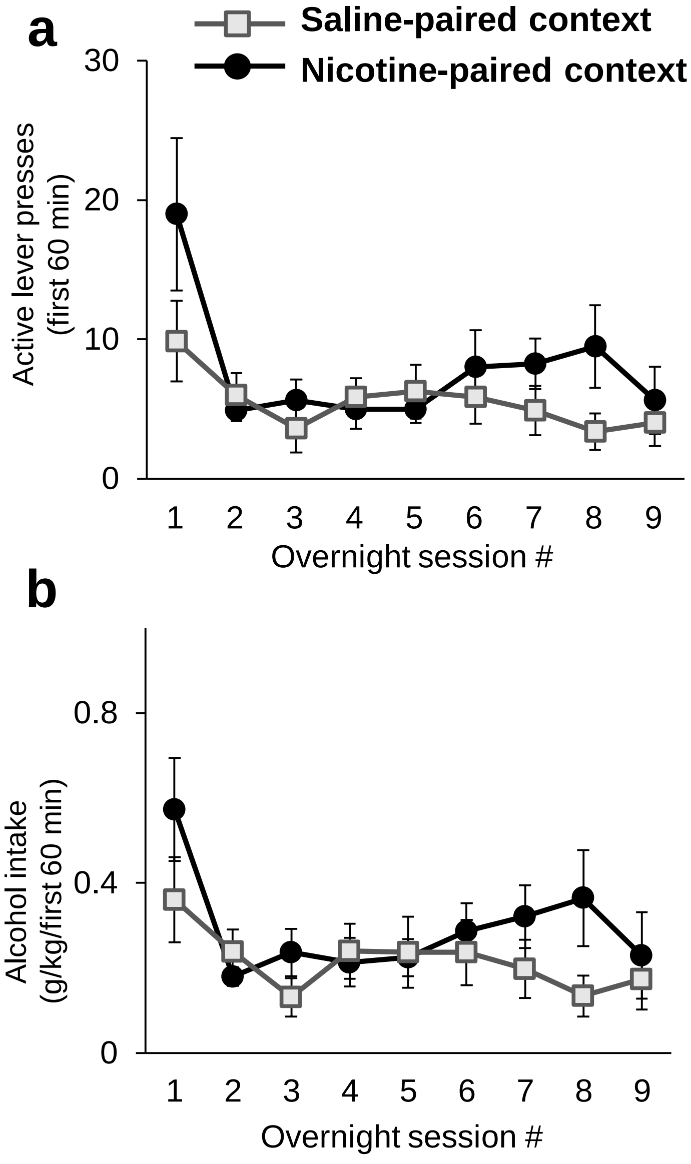

Figure S3. No effects of nicotine pre-treatment paired with the operant chamber or with the home cage on operant alcohol self-administration during the first hour of intermittent overnight sessions. a-b. Mean ± SEM number of active lever presses (a) and alcohol intake normalized to body weight (b) during the first 60 min of overnight sessions. Mixed-model ANOVA; A main effect of Session for active-lever presses [$F(8,112)=4.55$, $p<0.05$] and for alcohol intake [$F(8,112)=3.64$, $p<0.05$], but no significant main effect of Context-pairing and no significant Context-pairing X Session interaction (all p's>0.05). n=8 per group.

Figure S4, Zipori et al.

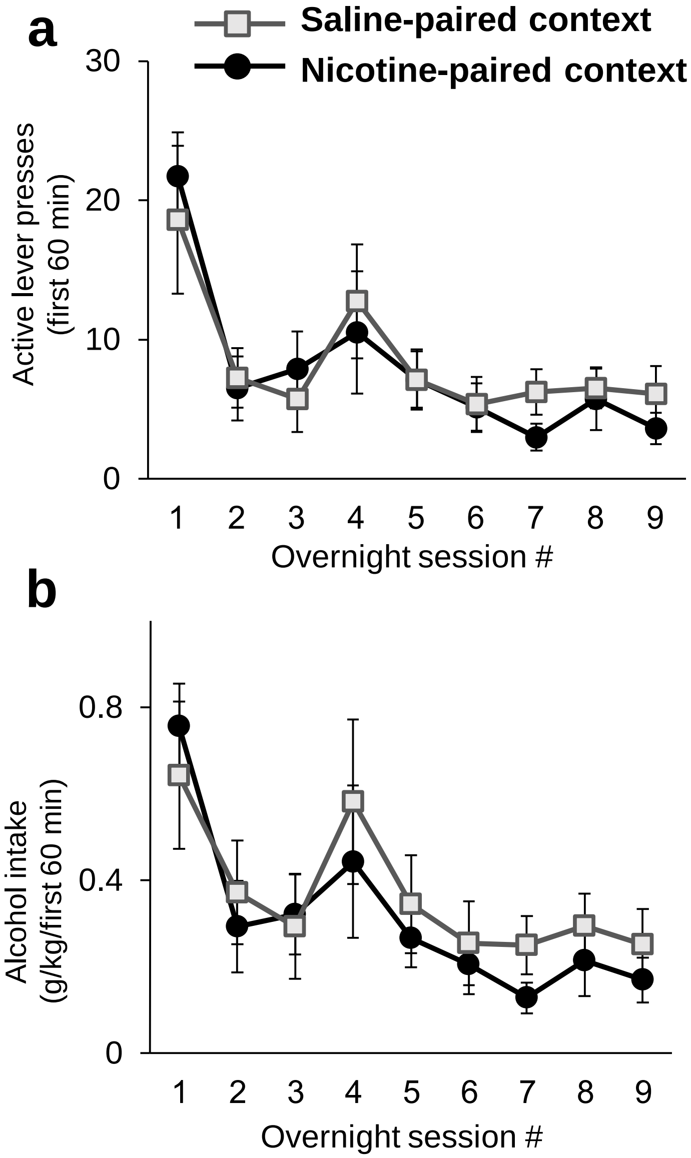

Figure S4. No effects of nicotine pre-treatment paired with the operant chamber or with a different context on operant alcohol self-administration during the first hour of intermittent overnight sessions. a-b. Mean ± SEM of the number of active lever presses (a) and alcohol intake normalized to body weight (b) during the first 60 min of overnight sessions. Mixed-model ANOVA; A main effect of Session for active-lever presses [$F(8,112)=9.35$, $p<0.05$] and for alcohol intake [$F(8,112)=7.29$, $p<0.05$], but no significant main effect of Context-pairing and no significant Context-pairing X Session interaction (all p's>0.05). n=8 per group.

Figure S5, Zipori et al.

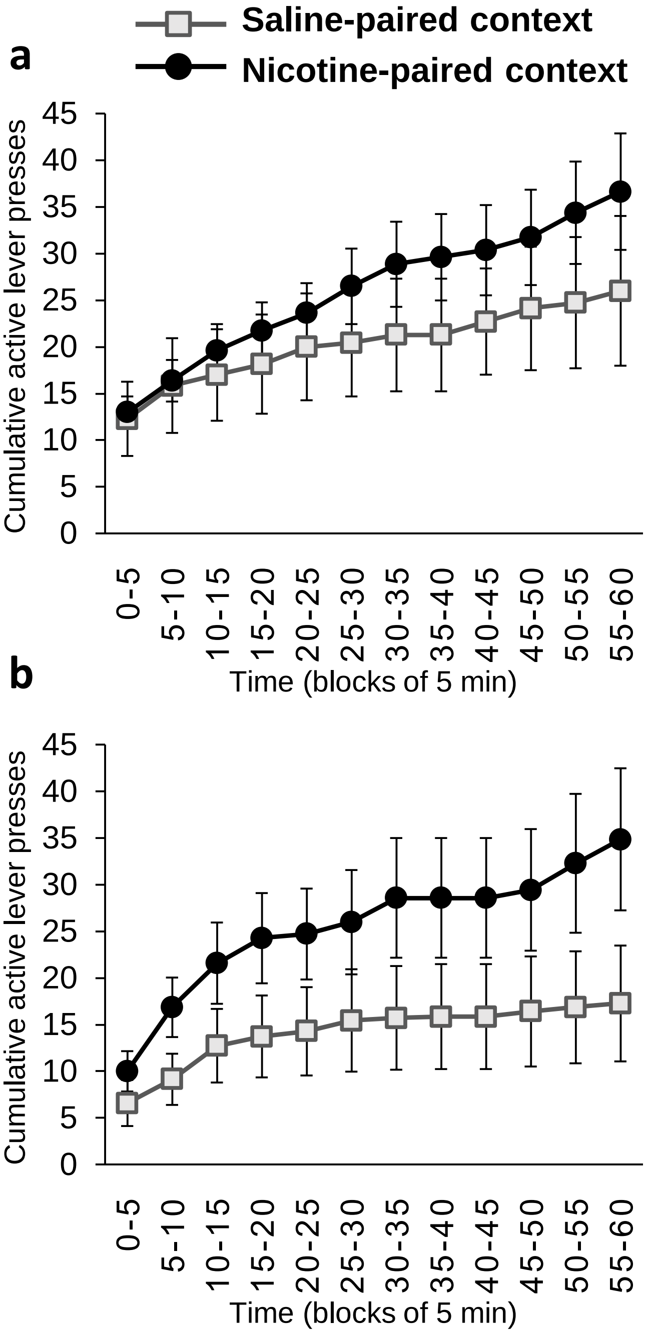

Figure S5. Within-session pattern of operant behavior after abstinence in extinction and reacquisition. Cumulative mean ± SEM of the number of active lever presses in bins of 5 min, indicative of the rate of: **a.** Alcohol seeking during the extinction session. **b.** Alcohol self-administration during the reacquisition session. Mixed-model ANOVA, extinction: A main effect of Time-bin [$F(11,143)=16.54$, $p<0.001$], but no significant main effect of Context-pairing and no significant Context-pairing X Time-bin interaction (p's>0.05). Reacquisition: a main effect of Time-bin [$F(11,143)=14.30$, $p<0.001$], marginally significant main effect of Context-pairing [$F(1,13)=3.55$, $p=0.08$], and a Context-pairing X Time-bin interaction [$F(11,143)=2.18$, $p<0.05$]. n=7-8 per group.
